# Utilization of Optical Flow Algorithms to Monitor Development of Tail Biting Outbreaks in Pigs

**DOI:** 10.3390/ani10020323

**Published:** 2020-02-18

**Authors:** Yuzhi Z. Li, Lee J. Johnston, Marian S. Dawkins

**Affiliations:** 1 West Central Research and Outreach Center, University of Minnesota, Morris, MN 56267, USA; johnstlj@umn.edu; 2 Department of Zoology, University of Oxford, John Krebs Field Station, Wytham, Oxford OX2 8QJ, UK; marian.dawkins@zoo.ox.ac.uk

**Keywords:** behavior, optical flow, pigs, tail biting

## Abstract

**Simple Summary:**

Optical flow is a measurement of movement of individual objects in a group and can be used to monitor activity changes in both humans and animals. Using optical flow to monitor activity changes in pigs has not yet been reported. In this study, behavior of pigs in four pens of 30 pigs was video-recorded. The video-recordings before and during the first outbreak of tail biting were viewed manually to register active and resting behaviors of pigs. The same video-segments for behavioral evaluation were used for calculation of optical flow. Results indicate that mean optical flow was higher three days before and during the day of the tail-biting outbreak, suggesting increased activity level, compared to 10 days before the outbreak. All optical flow measures were correlated with time spent standing by pigs, indicating that movement during standing was associated with optical flow measures. These results suggest that optical flow measures might be a useful tool for automatically detecting activity changes associated with onset of tail-biting outbreaks.

**Abstract:**

A study was conducted to evaluate activity changes in pigs associated with the development of tail-biting outbreaks using optical flow algorithms. Pigs (*n* = 120; initial body weight = 25 ± 2.9 kg) housed in four pens of 30 pigs were studied for 13 weeks. Outbreaks of tail biting were registered through daily observations. Behavior of pigs in each pen was video-recorded. Three one-hour video segments, representing morning, noon, and afternoon on days 10, 7, and 3 before and during the first outbreak of tail biting were scanned at 5-min intervals to estimate time budget for lying, standing, eating, drinking, pig-directed behavior, and tail biting. The same video segments were analyzed for optical flow. Mean optical flow was higher three days before and during the tail-biting outbreak, compared to 10 days before the outbreak (*p* < 0.05), suggesting that pigs may increase their activity three days before tail-biting outbreaks. All optical flow measures (mean, variance, skewness, and kurtosis) were correlated (all *p* < 0.01) with time spent standing, indicating that movement during standing may be associated with optical flow measures. These results suggest that optical flow might be a promising tool for automatically monitoring activity changes to predict tail-biting outbreaks in pigs.

## 1. Introduction

Tail biting is a common problem in swine production and can cause considerable economic losses to pork producers and major welfare complications for pigs [1,2,3]. While tail biting is considered an abnormal behavior due to stress and frustration in the pig [4,5], it usually occurs sporadically [6,7]. To prevent tail biting, pork producers in the U.S. and some European countries usually dock the tails of pigs within the first few days after birth due to a lack of sensible alternatives. Since tail docking is a painful procedure for the pig [8,9], some countries prohibit tail docking for animal welfare reasons [10]. Although tail docking is allowed in the U.S., pork producers are interested in alternatives to tail docking that could prevent pigs from tail biting. Predicting outbreaks of tail biting through early signs displayed by pigs can be a first step toward development of measures that prevent tail biting. Previous researchers [11,12] suggested that pigs increase their activity level days before outbreaks of tail biting. If proven, changes in activity level could be used to predict outbreaks of tail biting [13]. However, prediction of tail biting through monitoring activity changes requires observation of pigs for extended periods of time (e.g., weeks), which is time-consuming and not practical. Optical flow algorithms can monitor activity changes in animals automatically over an extended period [14]. So, optical flow algorithms potentially could be used to predict outbreaks of tail biting in pigs. Based on previous studies, we hypothesize that mean optical flow would increase before an outbreak of tail biting.

Optical flow is measured as the rate of change in brightness in different parts of a moving image [15,16]. Optical flow measures have been used to monitor activities of broiler chickens [14,17] and ducks [18] in large groups. Dawkins et al. [14,17] have demonstrated that optical flow measures were correlated with gait score, hock burn, and mortality in broiler chickens, suggesting that optical flow is a useful tool to monitor animal welfare automatically on commercial farms. Likewise, Li et al. [18] reported that ducks housed with high stocking density had lower mean optical flow compared with ducks penned with low stocking density, suggesting that high stocking density reduced activity of ducks. However, optical flow has not yet been explored for monitoring activities of pigs. In this study, we used optical flow algorithms to evaluate activity changes in pigs associated with onset of tail-biting outbreaks.

## 2. Materials and Methods 

The Institutional Animal Care and Use Committee of the University of Minnesota reviewed and approved the protocol for this study (IACUC # 1412-032165A). 

### 2.1. Animals, Housing, and Management

This study was part of a larger project [19] conducted at the University of Minnesota’s West Central Research and Outreach Center in Morris, Minnesota that investigated effects of tail docking on tail biting in pigs. In the larger study, pigs (Landrace × Yorkshire × Duroc; *n* = 240) with equal numbers of gilts and barrows were housed in a windowless confinement growing-finishing barn. Half of the pigs, with the sex balanced, were tail docked at birth, and remaining pigs maintained intact tails. The windowless barn had 4 pens (4.7 m × 4.6 m) arranged in a row along an outside wall on either side of a central alleyway (8 pens total). The layout of pens was identical. Pigs with docked tails were allocated to 4 pens, and pigs with intact tails were allocated to another 4 pens (30 pigs/pen) in both rows of pens in an alternating arrangement. The front gate and partitions for all pens were constructed of solid PVC (polyvinyl chloride, a synthetic thermoplastic material made by polymerizing vinyl chloride) planking (white in color) so that pigs in each pen could not see pigs in other pens. Pigs were sorted by tail docking (docked or intact), weight, and sex. However, due to the required time of videotaping ahead of spontaneous outbreaks of tail biting, only 120 pigs were included in data analysis for this current study. Animal housing and management protocol from birth to commencement of the study has been described previously [19]. Animal management related to this study is described herein.

At 9 weeks of age, pigs that had no signs of tail damage were selected for the study. Four pens of 30 pigs (n = 120; initial weight = 25 ± 2.9 kg) that had their first tail-biting outbreak on Monday or Friday were included for data collection. Among the 4 pens, two pens housed pigs with docked tails, and the other two pens housed pigs with tails intact. Tails were docked 1.3 to 2.5 cm from the base of the tail using sharp, side-cutting pliers. Pigs were mixed at 9 weeks, and pen groups were formed with equal numbers of barrows and gilts. Mean weight and within pen variation (coefficient of variation) across all pens were similar. Pigs remained in their designated pens for 16 weeks until pigs were 25 weeks of age with average body weights of 127 ± 9.6 kg. 

Each pen was equipped with a dry feeder (3 feeding spaces/feeder) and two nipple drinkers on fully-slatted floors. Three feeding spaces for a group of 30 pigs are considered standard practice for confinement systems in North America [20,21,22]. Floor space allowance was 0.72 m^2^/pig throughout the study period, excluding the space occupied by the feeder. All pigs had free access to water and corn-soybean meal-based diets in mash form formulated according to NRC recommendations [23]. A computerized controller and a mechanical ventilation system maintained air quality and room temperatures as close to recommended targets as possible in the barn [24]. Room temperature at the floor level was recorded at 15-min intervals by a Hobo Data Logger (HOBO Pro-V2, Tem/RH, Onset, Bourne, MA, USA). During the study period, average room temperature was 25.7 ± 1.54 °C (range = 21.7 to 30.0 °C). Lights in the barn were on for 8 h daily starting from 0800 h. Pigs were checked at least twice daily to ensure that pigs had free access to feed and water and had no signs of illness, injury, or tail damage. No environmental enrichment or rooting materials were provided throughout the study to be consistent with practices in confinement production systems in North America.

### 2.2. Data Collection

#### 2.2.1. Outbreaks of Tail Biting and Tail Damage 

All pigs were checked for visible blood on their tails through routine daily observations inside the pen. Once a pig with visible blood on the tail appeared in a pen, all pigs in the pen were assessed for tail damage. Tail damage was evaluated using a modified 0 to 4 scoring system based on the method of Kritas and Morrison [2]: score 0 = no damage, score 1 = healed lesions with scars or small scabs, score 2 = evidence of chewing or puncture wounds with visible blood but no signs of infection, score 3 = evidence of chewing or puncture wounds with visible blood and signs of infection or partial loss of the tail without signs of infection, and score 4 = partial or total loss of the tail with signs of infection. When at least two pigs with a tail score of 2, or at least one pig with a tail score of 3, were identified in a pen, the pen was considered to have an outbreak of tail biting. Details on tail score assessments were described previously [19]. Pigs that sustained tail scores of 3 or 4 for more than 24 h were removed to hospital pens for recovery. Since data collection for this paper ended on the day of the first tail-biting outbreak, no pigs were removed.

#### 2.2.2. Behavior and Optical Flow

Pigs were allowed one week for adaptation to the new environment in the growing-finishing barn. Then, behavior of pigs was video-recorded two days (Monday and Friday) weekly when pigs were between 10 and 23 weeks of age. Each pen was fitted with an infrared digital camera (TruVision High Definition TVI Bullet Camera TVB-4403, Interlogix, Costa Mesa, CA, USA) mounted on the wall of the barn. Each camera was mounted at a height of 2.2 m above the floor with an angle of 30 degrees downward from the wall. Cameras covered about 90% of the floor area of each pen. The cameras were connected to a computer equipped with a time-lapse DVR device and video-recording software (Geo Vision Multicam Digital Surveillance System V8.2; USA Vision Systems Inc., Irvine, CA, USA). On video-recording days, each pen was recorded for 8 h starting from 0830 h. Among the 8 h of video-recording each day, 3 h of videos were used for behavior and optical flow analysis. The three hours of video consisted of 1 h each in the morning (0900 h to 1000 h), noon (1200 h to 1300 h), and afternoon (1500 h to 1600 h).

Optical flow for pigs in each pen was analyzed at the University of Oxford, Oxford, UK using methods described previously by Dawkins et al. [17,25]. Optical flow measures apparent rate of change of image brightness and so is particularly suitable for detecting movement. The computer software captured 14,400 image frames per hour (4 frames/sec). Each frame of the video was divided into 1200 8 × 8 pixel blocks. The optical flow algorithm estimated, for each block, the rate of changes of image brightness between two consecutive image frames and then took the spatial mean of all blocks over that time period. Initially, optical flow measures (mean, variance, skewness, and kurtosis) over each minute were averaged for each pen. Outliers of the average were identified when variance of optical flow was 3 times greater than the mean variance for the hour. When outliers were identified, original video clips were checked. In most cases, outliers were caused by either poor quality of the video (probably due to either bad connection of cables or electronic interference) or a fly on the lens of a camera. When outliers were confirmed, all four optical flow measures (mean, variance, skewness, and kurtosis) during that time period were excluded from the analysis. About 1% of the optical flow data were deemed outliers and removed. 

To evaluate relationship between optical flow measures and activity of pigs, the same 3 h of video used for optical flow analysis were scanned manually to estimate behavioral time budget. Behaviors of interest included resting (lying recumbent laterally or sternally); standing (four legs upright still, walking, or exploring the pen or floor); eating (pig’s head in the feeder); drinking (pig’s snout touching the drinker); pig-directed behavior (nosing, rooting, chewing, or pushing any part of the body other than the tail of another pig); and tail biting (nosing, rooting, or chewing the tail of another pig), as described previously [11,26,27]. These behaviors were considered exclusive. For instance, when a pig was nosing any body part other than the tail of a penmate, the pig was considered performing pig-directed behavior, regardless of whether the pig was standing or lying while performing the behavior. The video segments were scanned at 5-min intervals. At each scan, the number of pigs performing behaviors of interest in each pen was recorded. Time spent on each behavior of interest was calculated by the number of pigs performing the behavior expressed as a percentage of total number of pigs in the view [28].

#### 2.2.3. Data Analysis 

Only video-recordings 10 days in advance of the very first tail-biting outbreak for a pen were used in the data analysis. Video-recordings ahead of subsequent tail-biting outbreaks for a given pen were not considered. Proper evaluation of the optical flow algorithms required optical flow measurements and assessments of pig behavior from video-recordings on the same day in advance of spontaneous tail-biting outbreaks. The video-recording schedule was set (Monday and Friday weekly) to accommodate requirements of the larger tail-biting project [19]. Based on this video-recording schedule, four days relative to the first tail-biting outbreak were defined as: (1) day 10 or 11 before the outbreak, (2) day 7 before the outbreak, (3) day 3 or day 4 before the outbreak, and (4) the day of the outbreak. Four of the eight pens (2 pens with docked tails and 2 pens with intact tails) assigned to the large project satisfied these requirements and were included in the final analysis for the study described herein. For pigs that had the first outbreak on a Monday, the four days represented day 10, day 7, and day 3 before and on the day of the first outbreak. Likewise, for pigs that had the first outbreak on a Friday, the four days represented day 11, day 7, and day 4 before and on the day of the first outbreak. For data analysis purposes, data from day 10 and day 11 before the first outbreak were considered as the same period and referred to as day 10 before the first outbreak. Similarly, data from day 3 and day 4 before the first outbreak were referred as day 3 before the first outbreak in the statistical model. Through the rest of this paper, the four days relative to the first outbreak of tail biting are defined as day 10, day 7, and day 3 before and on the day of the first outbreak.

Data were analyzed using the Glimmix and correlation procedures of the SAS software (version 9.4) [29]. The Glimmix model included day relative to the first outbreak of tail biting (day 10, day 7, and day 3 before and on the day of the outbreak); time of the day (morning, noon, and afternoon); and their interaction as fixed effects, with the pen serving as the experimental unit. Due to the small sample size, tail docking was not included in the model. Dependent variables included optical flow measures and behavioral time budget. Differences among means were tested by the adjusted Tukey’s test for multiple levels. All tests were two-tailed tests. Data are reported as least-squared means and standard errors, and significant differences were identified at *p* < 0.05 and trends at *p* < 0.10.

Correlation among optical flow measures and behavioral time budget was examined using the correlation procedure with the Pearson coefficient. For correlation analysis, raw data were averaged over each hour for each video-recording day for each pen (number of observations = 48; 4 pens × 4 d/pen × 3 h/d) and then used in the correlation model. Correlation was identified at *p* < 0.05 and trends at *p* < 0.10 for Pearson coefficients.

## 3. Results 

### 3.1. Number of Tail-Biting Outbreaks Observed

For the four pens that were included in the data analysis for this paper, the first outbreak of tail biting occurred when pigs were 16 weeks (one pen with intact pigs); 17 weeks (one pen with docked pigs); and 18 weeks (two pens, one with intact pigs and one with docked pigs) of age. Two pens had the first outbreak on Mondays (16 weeks and 18 weeks of age), and the other two pens had the first outbreak on Fridays (17 weeks and 18 weeks of age).

### 3.2. Effects of Day Relative to the First Outbreak of Tail Biting

Mean optical flow for pigs was higher (F_3,9_ = 12.10; *p* < 0.002) 3 d before and on the day of the first tail-biting outbreak compared to 10 d before the outbreak (Table 1). Mean optical flow 7 d before the first outbreak was not different from other days. Variance of optical flow was higher 3 d before and on the day of the first tail-biting outbreak compared with 10 d and 7 d before the outbreak (F_3,9_ = 18.61; *p* = 0.001). Skewness (F_3,9_ = 12.10; *p* = 0.002) and kurtosis (F_3,9_ = 17.10; *p* = 0.001) of optical flow were higher 10 d before a tail-biting outbreak but declined by 7 d before the outbreak and remained low thereafter.

On the day of the first tail-biting outbreak, pigs tended to spend less time standing (F_3,9_ = 2.89; *p* = 0.09) compared to day 3 before the outbreak. Pigs tended to spend more time lying (F_3,9_ = 2.84; *p* = 0.10) on the day of the first outbreak compared to day 10 before the outbreak. In addition, pigs spent less time drinking (F_3,9_ = 3.85; *p* = 0.05) on the day of the first outbreak than day 10 before the outbreak. Time budget for eating, pig-directed behavior, and tail biting did not change across the four observation days.

### 3.3. Effects of Time of Day (Morning, Noon, and Afternoon)

Mean optical flow was higher (F_2,6_ = 15.46; *p* = 0.01) in the morning and at noon than in the afternoon (Table 2). Likewise, variance of optical flow was higher in the morning and at noon than in the afternoon (F_2,6_ = 16.77; *p* = 0.01). On the other hand, skewness and kurtosis were higher in the morning and afternoon than at noon (*p* = 0.02).

Pigs tended to spend more time (F_2,6_ = 4.67; p = 0.06) standing in the morning and at noon than in the afternoon. In addition, pigs spent more time eating in the morning than at noon (F_2,6_ = 5.87; *p* = 0.04). Time of day did not affect time spent by pigs lying, drinking, performing pig-directed behaviors, or tail biting.

**Figure 1:**
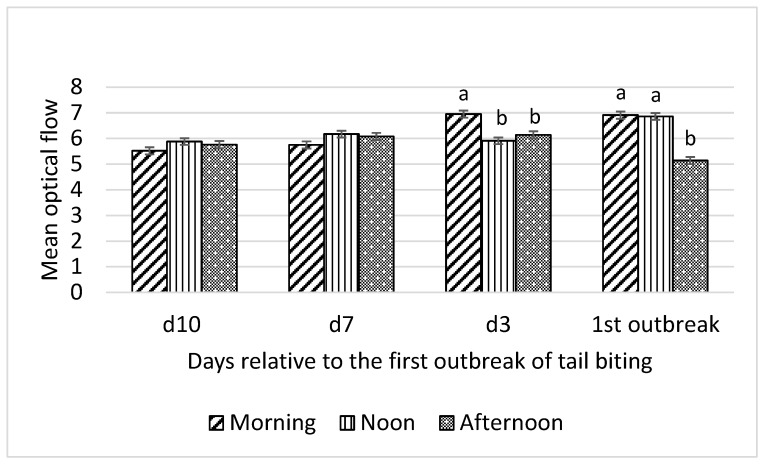
Interaction between day (10 d, 7 d, and 3 d before and during) relative to the first outbreak of tail biting and time of day (morning, noon, and afternoon) for mean optical flow; ^ab^—within days means without a common superscript differ (*p* < 0.01).

**Figure 2:**
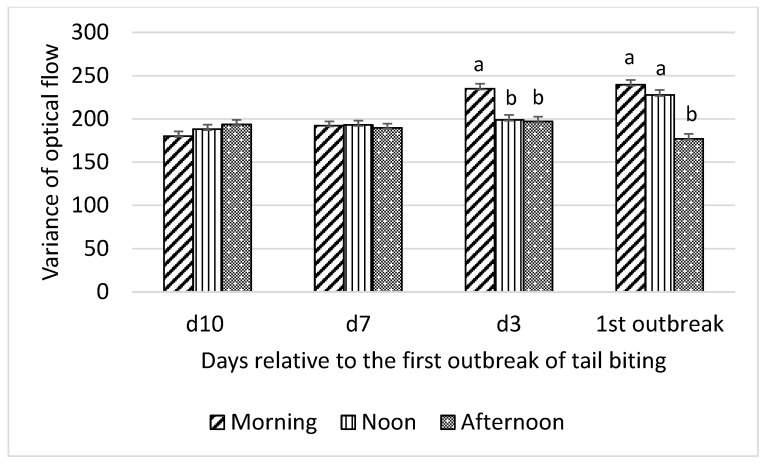
Interaction between day (10 d, 7 d, and 3 d before and during) relative to the first outbreak of tail biting and time of day (morning, noon, and afternoon) for variance of optical flow; ^ab^—within days means without a common superscript differ (*p* < 0.01).

**Figure 3:**
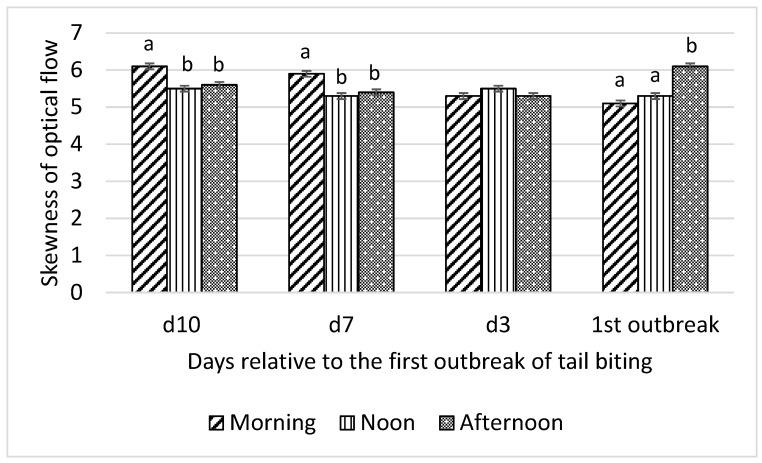
Interaction between day (10 d, 7 d, and 3 d before and during) relative to the first outbreak of tail biting and time of day (morning, noon, and afternoon) for skewness of optical flow; ^ab^—within days means without a common superscript differ (*p* < 0.01).

**Figure 4:**
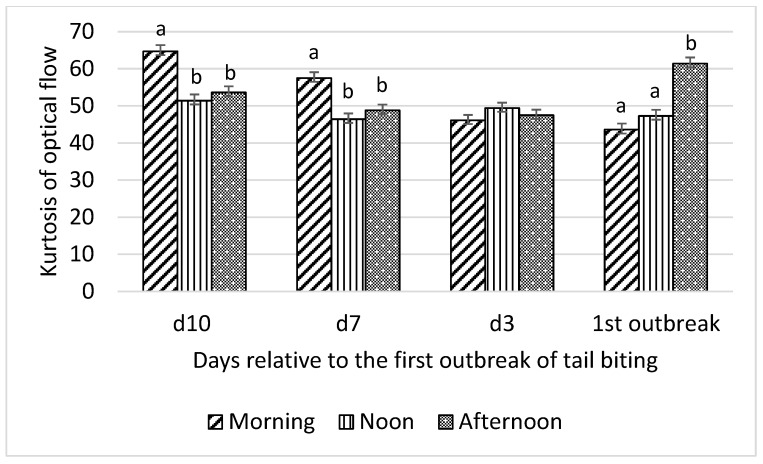
Interaction between day (10 d, 7 d, and 3 d before and during) relative to the first outbreak of tail biting and time of day (morning, noon, and afternoon) for kurtosis of optical flow; ^ab^—within days means without a common superscript differ (*p* < 0.01).

Interactions between time of day and day relative to the first tail-biting outbreak were detected for all optical flow measures (F_6,18_ = 13.01 to 22.31; all *p* < 0.001) and time spent by pigs standing (F_6,18_ = 4.46; *p* = 0.01), lying (F_6,18_ = 3.46; *p* = 0.02), and eating (F_6,18_ = 3.22; *p* = 0.03). Consequently, effects of time of day on these variables were examined for each day relative to the first outbreak and are presented in Figure 1, Figure 2, Figure 3 and Figure 4. 

Mean optical flow was not different among morning, noon, and afternoon observation periods on day 10 and day 7 before the first tail-biting outbreak (Figure 1). On day 3 before the first outbreak, mean optical flow was higher (*p* < 0.01) in the morning than at noon and in the afternoon. Furthermore, on the day of the first tail-biting outbreak, mean optical flow was higher (*p* < 0.01) in the morning and at noon than in the afternoon.

Variance of optical flow showed the same pattern of interaction between time of day and day relative to the first tail-biting outbreak, as observed for mean optical flow (Figure 2).

Skewness of optical flow was higher in the morning than at noon and in the afternoon on day 10 and day 7 before the tail-biting outbreak and higher in the afternoon than at noon and in the morning on the day of the outbreak (all *p* < 0.01; Figure 3). No difference in skewness of optical flow among morning, noon, and afternoon on day 3 before the outbreak was observed. Kurtosis of optical flow showed the same pattern of interaction between time of day and day relative to the first tail-biting outbreak as skewness of optical flow (Figure 4).

**Figure 5:**
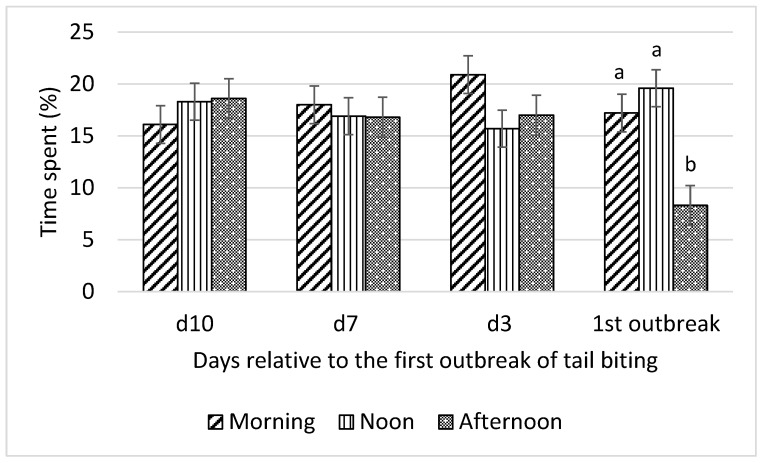
Interaction between day (10 d, 7 d, and 3 d before and during) relative to the first outbreak of tail biting and time of day (morning, noon, and afternoon) for time spent standing (% of observation time); ^ab^—within days means without a common superscript differ (*p* < 0.01).

Prior to the first tail-biting outbreak, no difference in time spent standing among morning, noon, and afternoon was observed (Figure 5). However, on the day of the first tail-biting outbreak, pigs spent more time (*p* < 0.01) standing in the morning and at noon than in the afternoon. This pattern on the day of the outbreak was also observed for mean optical flow data. 

**Figure 6:**
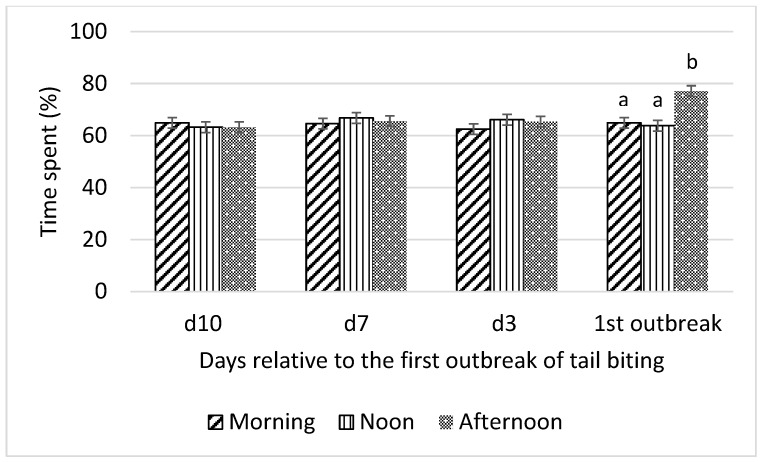
Interaction between day (10 d, 7 d, and 3 d before and during) relative to the first outbreak of tail biting and time of day (morning, noon, and afternoon) for time spent lying (% of observation time); ^ab^—within days means without a common superscript differ (*p* < 0.01).

Pigs spent less time lying (*p* < 0.01; Figure 6) in the morning and at noon than in the afternoon on the day of the first tail-biting outbreak. This corresponds to the results of high mean optical flow observed (Figure 1) and more time spent standing (Figure 5) in the morning and at noon than in the afternoon on the day of the first outbreak. No difference in time spent lying among morning, noon, and afternoon was observed on any day prior to the outbreak.

**Figure 7:**
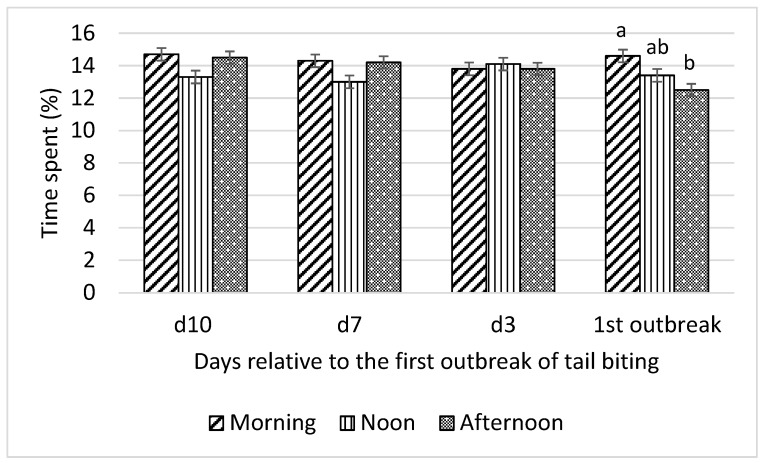
Interaction between day (10 d, 7 d, and 3 d before and during) relative to the first outbreak of tail biting and time of day (morning, noon, and afternoon) for time spent eating (% of observation time); ^ab^—within days means without a common superscript differ (*p* < 0.05).

On the day of the first tail-biting outbreak, pigs spent more time eating (*p* < 0.05; Figure 7) in the morning than in the afternoon. No difference in time spent eating was observed among morning, noon, and afternoon on any day before the outbreak.

### 3.4. Correlation between Optical Flow Measures and Behavioral Time Budget

Mean optical flow and variance of optical flow were correlated positively with time spent standing (*p* < 0.01; *n* = 48; Table 3). In addition, skewness and kurtosis of optical flow were negatively correlated with time spent standing (*p* < 0.01). For pig-directed behavior, a positive correlation between variance of optical flow and time spent performing pig-directed behavior was detected (*p* < 0.05), and a tendency for a positive correlation between mean optical flow and time spent performing pig-directed behavior was detected (*p* < 0.10). Optical flow measures were not correlated with time spent tail biting, except that variance of optical flow tended to be negatively correlated (*p* < 0.10) with time spent tail biting.

## 4. Discussion

To our knowledge, this is the first published study that monitors changes in activity level of pigs using optical flow measures to predict onset of tail-biting outbreaks. This study was conducted based on findings from previous studies [11,12,30] that indicate pigs increased their activity level before tail-biting outbreaks. If this observation is true consistently, outbreaks of tail biting may be predictable through monitoring of activity changes in pigs [13]. In previous studies, the ”increased activity” refers to increased time spent standing [11] and sitting, increased posture changes [12], increased aggression and manipulative behavior [30], and decreased time spent lying inactive [11,12]. We assumed that changes in standing, aggression, and pig-directed behavior would result in changes in movements of pigs, which could be detected by optical flow measures.

As expected, in the current study, we observed that mean optical flow increased on day 3 before and on the day of the first tail-biting outbreak compared to day 10 before the outbreak, which suggests that pigs may have increased their level of activity 3 d before the tail-biting outbreak. We did not detect a difference in mean optical flow between day 10 and day 7 before the tail-biting outbreak, which suggests that day 7 before the outbreak might be too early to detect activity changes for predicting tail-biting outbreaks. These results support results of previous studies [11,12]. Statham et al. [11] reported that pigs in tail-biting pens were more active, with increased time spent standing and decreased time spent lying inactive 4 d before a tail-biting outbreak compared with pigs in non-tail-biting pens. Likewise, Zonderland et al. [12] reported that pigs increased their activity level, as indicated by increased tail biting, ear biting, sitting, posture changes and lying ventrally, and decreased lying inactive immediately before a tail-biting outbreak, compared with 6 d before the outbreak. In contrast, Wedin et al. [31] did not find differences in standing, sitting, lying laterally, or lying ventrally between the outbreak group and control group from 7 d to 1 d before an outbreak of tail biting. Instead, they [28] observed a higher percentage of pigs with uncurled tails in tail-biting pens the day before a tail-biting outbreak than in control pens. They surmised that tail posture is a better indicator for predicting tail-biting outbreaks than activity changes. 

In general, changes in variance of optical flow across 4 d relative to the outbreak of tail biting showed a similar pattern as mean optical flow in the current study. Variance of optical flow increased on day 3 before and on the day of the tail-biting outbreak compared to day 10 and day 7 before the outbreak. Increased variance of optical flow could be due to either increased or decreased movement of some individuals within a group. For instance, in a study with ducks, Li et al. [18] reported that ducks housed under high stocking density had lower mean optical flow but higher variance of optical flow than ducks housed under low stocking density. The authors explained that high stocking density may not only limit activity of ducks, as indicated by low mean optical flow, but may also increase the incidence of lameness in some ducks, resulting in slow or abnormal gait of these ducks. The authors speculated that decreased movement of these lame ducks contributed to high variance of optical flow in high stocking density groups compared to ducks in low density groups. In the current study, the increased variance of optical flow was associated with increased mean optical flow, indicating that the increased variance of optical flow was due to increased movement of some but not all individuals within a group. In other words, the increased activity at the pen level could have resulted from increased activity of some pigs but not all pigs in the pen. We speculate that these individual pigs with increased activity 3 days before the tail-biting outbreak could be potential tail biters or victim pigs due to either excitement [30] or discomfort [12]. There is evidence [11,12,30] that tail biters, victim pigs, and control pigs (neither biters or victims) in the same pen could behave differently during the development of a tail-biting outbreak. Tail biters and victim pigs may be more active than control pigs, which may contribute to increased variance of optical flow. Due to the limited scope of the current study, we could not identify individual pigs. Further research would be needed to track activity of individual pigs automatically during the development of tail-biting outbreaks to gain a more thorough understanding of variance in optical flow. 

Changes in skewness and kurtosis of optical flow over four days relative to the tail-biting outbreak showed a similar pattern. Both skewness and kurtosis decreased on day 7 and day 3 before and on the day of a tail-biting outbreak, compared to day 10 before the outbreak. According to Dawkins et al. [17], highly positive skewness of optical flow means “movement mode displaced from the mean in the direction of slow movement” and highly positive kurtosis of optical flow represents “unusual movement”. Dawkins et al. [17] reported that broiler flocks with healthy birds had lower skewness and kurtosis of optical flow than flocks with lame birds. The authors explained that healthy birds walked “in the same way” and lame birds walk with “various degrees of difficulty”, resulting in mixed ability of walking or mixed movement of individuals within the group, which is reflected in high skewness and kurtosis of optical flow. Applying the same principle to explain our data, we assume that low skewness and kurtosis indicate movement mode displaced close to the mean in the direction of fast movement. In other words, pigs that increased their activity moved “in the same way without difficulty”. These data further confirm that some pigs in a pen increased their activity before tail-biting outbreaks.

In general, time spent standing defines active pigs [11,31]. As expected, mean optical flow was correlated positively with time spent standing in the current study, suggesting that optical flow was increased as activity level of pigs increased. Variance of optical flow was also correlated positively with time spent standing, indicating that individual pig variance in activity was increased. 

The negative correlation between skewness of optical flow and time spent standing in the current study may be translated that movement of pigs was getting faster as time spent standing was increased [17]. A reasonable assumption is that time spent standing increased as pigs became more active [31]. The increased activity level in a pen may be associated with some pigs moving faster than others in the pen, resulting in a negative correlation between kurtosis of optical flow and time spent standing [17].

A positive correlation between variance of optical flow and time spent performing pig-directed behaviors was detected in the current study. This may indicate that some pigs performed more pig-directed behaviors than others, which increased individual variation of movement of pigs in a pen.

In the current study, we did not collect data on posture changes, lying active, lying inactive, aggression, or sitting. Initially, time spent sitting was recorded, but due to low incidence, it was combined with time spent lying. Lying active and inactive is hard to distinguish from video-recordings, so they were not recorded separately. Aggression among pigs was very minimal in the current study, because the first tail-biting outbreak was observed 5 weeks after pigs were allocated to their pens. Consequently, dominance hierarchy was well-established by the time the first outbreak of tail biting occurred. We suggest future researchers record posture changes, lying active, lying inactive, and sitting postures, which may provide insight into why optical flow data and posture/behavior data were inconsistent in the current study.

In the current study, we studied the temporal pattern of activity and postures of pigs from 10 d before the first outbreak of tail biting. All pens included in data analysis had a tail-biting outbreak, and no control pens without tail-biting outbreaks were included. Control pens were not included, because there were only two pens among eight pens in the larger project that did not exhibit an outbreak of tail biting. Larsen et al. [13] suggested including contemporary control pens without tail biting when studying development of tail-biting outbreaks to minimize confounding effects of age. In general, pigs reduce their activity as they grow. Pedersen et al. [32] reported that, compared to 40-kg pigs, 80-kg pigs were less active. However, pigs may not change behavioral patterns within a short period of time, as short as 10 d in the current study. It is worthwhile to note that the number of pens (four pens) included in data collection for the current study is considered the minimum. Due to the unpredictability of tail-biting outbreaks, we could not include all pens (eight pens) originally designed for the study. Some pens had the first tail-biting outbreak too soon that no data were collected 10 d before the outbreak, while other pens had the first tail-biting outbreak on days that pigs were not video-recorded. Future research should include more pens for data collection and daily continuous behavior recording.

Optical flow measures are a complex outcome of the movements of individual objects and can be affected by both the objects and environmental factors [16]. Dawkins et al. [17,25] argued that mean optical flow is affected by body size of animals, light intensity, and distance between cameras and animals, so other measures of optical flow (variance, skewness, and kurtosis) should be considered in monitoring movements of animals. In the current study, pigs were housed in a windowless barn with artificial lights throughout the video recording periods. Light intensity was considered consistent throughout video-recording periods. However, we could not avoid changes in body weight/size during 10 d of video recording for each pen in the current study. With average daily gains of 944 g [19], the estimated weight change was about 9.4 kg for pigs during the video-recording period. It is not clear how body weight/size changes could affect optical flow measures in the current study. To address the issue of body weight/size changes, we included all optical flow measures in our analysis, as suggested by Dawkins et al. [17,25].

## 5. Conclusions

Results of this study indicate that pigs increased activity level 3 d before the first outbreak of tail biting. All optical flow measures (mean, variance, skewness, and kurtosis) were correlated with time spent standing, indicating that movement during standing was associated with optical flow measures. These results suggest that optical flow might be a promising tool for monitoring activity changes in pigs to predict onset of tail-biting outbreaks. Future research should investigate whether posture changes, lying active, and sitting affect optical flow measures are associated with onset of tail-biting outbreaks.

## Figures and Tables

**Table 1:** Optical flow measures and behavioral time budget for growing-finishing pigs in pens that experienced tail-biting outbreaks during the study period.

**Item**	**Days Relative to the First Outbreak of Tail Biting**				**SEM**	***p*-Value**
	**10 d Before**	**7 d Before**	**3 d Before**	**Outbreak**		
Number of pens ^1^	4	4	4	4		
Number of observations	12	12	12	12		
Optical flow measures:						
Mean	5.7 ^a^	6.0 ^ab^	6.3 ^b^	6.2 ^b^	0.08	0.002
Variance	187.2 ^a^	191.6 ^a^	209.6 ^b^	212.9 ^b^	3.06	0.001
Skewness	5.8 ^a^	5.5 ^b^	5.4 ^b^	5.5 ^b^	0.05	0.002
Kurtosis	56.3 ^a^	50.7 ^b^	47.6 ^b^	50.2 ^b^	0.93	0.001
Behavioral time budget ^2^, %:						
Standing	17.6 ^ef^	17.2 ^ef^	17.7 ^e^	14.1 ^f^	0.99	0.09
Lying	63.7 ^e^	65.6 ^ef^	64.7 ^ef^	68.3 ^f^	1.36	0.10
Eating	14.1	13.8	13.9	13.5	0.22	0.31
Drinking	2.6 ^a^	1.7 ^ab^	1.8 ^ab^	1.4 ^b^	0.29	0.05
Pig-directed	1.8	1.4	1.6	1.2	0.24	0.26
Tail biting	0	0.1	0	0.2	0.91	0.95

^1^—Among the four pens that experienced tail-biting outbreaks, two pens housed pigs with intact tails and two pens housed pigs with docked tails; ^2^—percentage of total observation time; ^ab^—means within a row without a common superscript differ (*p* < 0.05); and ^ef^—means within a row without a common superscript tend to differ (*p* < 0.10).

**Table 2:** Optical flow measures and behavioral time budget during morning, noon, and afternoon for growing-finishing pigs in pens that experienced tail-biting outbreaks during the study period ^1.^

**Item**	**Time of Day ^2^**			**SEM**	***p*-Value**	
	**Morning**	**Noon**	**Afternoon**		**Time of Day**	**Time of Day × Day Relative to Outbreak**
Number of pens ^3^	4	4	4			
Number of observations	16	16	16			
Optical flow measures:						
Mean	6.2 ^a^	6.2 ^a^	5.8 ^b^	0.07	0.01	<0.001
Variance	210.1 ^a^	201.4 ^a^	189.2 ^b^	2.63	0.01	<0.001
Skewness	5.6 ^a^	5.4 ^b^	5.6 ^a^	0.04	0.02	<0.001
Kurtosis	52.3 ^a^	48.6 ^b^	52.5 ^a^	0.77	0.02	<0.001
Behavioral time budget ^4^, %:						
Standing	18.0 ^e^	17.6 ^e^	14.5 ^f^	0.89	0.06	0.01
Lying	64.2	65.0	67.6	1.03	0.12	0.02
Eating	14.4 ^a^	13.5 ^b^	13.8 ^ab^	0.19	0.04	0.03
Drinking	1.6	2.2	1.7	0.24	0.21	0.66
Pig-directed	1.6	1.4	1.4	0.20	0.61	0.88
Tail biting	0.01	0.01	0	3.30	1.00	0.98

^1^—Data from 10, 7, and 3 days before and the day of the first outbreak of tail biting; ^2^—morning = 0900 to 1000 h, noon = 1200 to 1300 h, and afternoon = 1500 to 1600 h; ^3^—among the 4 pens that experienced tail-biting outbreaks, two pens housed pigs with intact tails and two pens housed pigs with docked tails; ^4^—percentage of total observation time; ^ab^—means within a row without a common superscript differ (*p* < 0.05); and ^ef^—means within a row without a common superscript tend to differ (*p* < 0.10).

**Table 3:** Pearson correlation coefficients between optical flow measures and behavioral time budget for growing-finishing pigs in 4 pens that experienced tail-biting outbreaks during the study period ^1^.

Item	Optical Flow Measures			
	Mean	Variance	Skewness	Kurtosis
Behavioral time budget ^2^, %:				
Standing	0.522 ***	0.356 ***	−0.506 ***	−0.462 ***
Pig-directed	0.254 *	0.286 **	−0.188	−0.172
Tail biting	−0.107	−0.252 *	−0.077	−0.087

^1^—n = 48 (hourly average of 4 d × 3 h/day × 4 pens; 4 d = 10, 7, and 3 d before and on the day of the first outbreak of tail biting); ^2^—percentage of total observation time; ***—*p* < 0.01; **—*p* < 0.05; and *—*p* < 0.10.
